# Hyper-Progressive Disease: The Potential Role and Consequences of T-Regulatory Cells Foiling Anti-PD-1 Cancer Immunotherapy

**DOI:** 10.3390/cancers13010048

**Published:** 2020-12-26

**Authors:** Christopher Tay, Yamin Qian, Shimon Sakaguchi

**Affiliations:** 1 Immunology Frontier Research Center, Department of Experimental Immunology, Osaka University, Yamadaoka, Suita, Osaka 565-0871, Japan; ctayjw@ifrec.osaka-u.ac.jp (C.T.); yaminq@ifrec.osaka-u.ac.jp (Y.Q.); 2 Laboratory of Experimental Immunology, Institute for Frontier Life and Medical Sciences, Kyoto University, Kyoto 606-8507, Japan

**Keywords:** cancer, Tregs, immunosuppression, PD-1, hyper-progressive disease

## Abstract

**Simple Summary:**

Since immune cells were found to attack cancer cells, strategies have been developed to sustain anti-cancer immune functions to prevent them from becoming exhausted in the midst of war. Immune checkpoint blockade therapy has gained significant traction with its ability to fast-track activation of immune cells as well as revive dormant immune cells. A prominent target for such treatment is programmed cell death protein 1 (PD-1). The natural role of PD-1 is in regulating the activity of immune cells, hence the term ‘checkpoint’. Blocking PD-1 can be effective especially when immune cells have easy access to cancer tissues. However, the immune cell population also contains cells that protect cancer cells from anti-cancer immune cells. One of these is the regulatory T-cell (Treg) subset, which is also regulated by PD-1. In this article, we review the downside of blocking PD-1 in Tregs when using PD-1 blockade therapy to treat cancer.

**Abstract:**

Antibody-mediated disruption of the programmed cell death protein 1 (PD-1) pathway has brought much success to the fight against cancer. Nevertheless, a significant proportion of patients respond poorly to anti-PD-1 treatment. Cases of accelerated and more aggressive forms of cancer following therapy have also been reported. Termed hyper-progressive disease (HPD), this phenomenon often results in fatality, thus requires urgent attention. Among possible causes of HPD, regulatory T-cells (Tregs) are of suspect due to their high expression of PD-1, which modulates Treg activity. Tregs are a subset of CD4^+^ T-cells that play a non-redundant role in the prevention of autoimmunity and is functionally dependent on the X chromosome-linked transcription factor FoxP3. In cancer, CD4^+^FoxP3^+^ Tregs migrate to tumors to suppress anti-tumor immune responses, allowing cancer cells to persist. Hence, Treg accumulation in tumors is associated with poor prognosis. In mice, the anti-tumor efficacy of anti-PD-1 can be enhanced by depleting Tregs. This suggests Tregs pose resistance to anti-PD-1 therapy. In this article, we review the relevant Treg functions that suppress tumor immunity and the potential effects anti-PD-1 could have on Tregs which are counter-productive to the treatment of cancer, occasionally causing HPD.

## 1. Introduction

Cancer treatment has made great strides in recent years due to the advent of immune checkpoint blockade therapy. More research is embracing the concept of reinvigorating cancer-killing T-cells inactivated by co-inhibitory molecules. Programmed cell death-1 (PD-1) is, by far, the target of choice given that its expression is closely associated with T-cell dormancy and exhaustion. Indeed, enhanced tumor rejection from combining anti-PD-1 and conventional therapies has been reported [1,2]. Nevertheless, there remains a significant percentage of non-responders. Much work is still required to address the shortcomings and even unintended consequences of using anti-PD-1 therapy. One example of the latter is hyper-progressive disease (HPD). 

HPD is a severe condition of cancer that is characterized by an acute acceleration in tumor growth following immunotherapy [3,4,5]. This was exemplified by a higher incidence of HPD among non-small cell lung cancer patients who received anti-PD-1 treatment (13.8%) compared to chemotherapy (5.1%) [6]. Although the overall rate of HPD occurrence seems to be fairly low at 10%, this varies widely with the type of cancer, ranging from an average of 9% in melanoma and others to 29% in head and neck squamous cell carcinoma. This partly stems from a lack of universal criteria for diagnosing HPD. Current methods differ on various factors such as tumor variable (diameter versus volume), rate of tumor growth (1.2 to 2 fold) and cut-off time to treatment failure [4,5,7]. There is, however, a clear consensus that HPD is an existential threat among anti-PD-1 treated cancer patients and that it often leads to a fatal outcome.

As more data become available, reasons behind this phenomenon would come to light and allow pre-emptive measures to be taken to mitigate HPD. Meanwhile, *MDM2* and *EGFR* mutations have been flagged as potential risks rather than culprits, on the account that the oncogenic nature of these gene alterations predispose tumors to progress [8,9]. Hence, current efforts are dedicated to determine the exact cause of anti-PD-1-induced HPD. To date, two mechanisms have been proposed. The first involves the Fc region of anti-PD-1 antibody instigating M2-like differentiation of tumor-associated macrophages, thus cultivating immunosuppressive conditions in the tumor [10]. The second concerns regulatory T-cells (Tregs), the focus of this review. 

Tregs are major perpetrators of cancer. Yet, there is considerable apprehension over the usage of Treg-targeted immunotherapy. This mainly arises from risks of adverse autoimmune reactions. For anti-PD-1 therapy, perhaps a calibrated approach would be needed as it is becoming evident that Tregs may not only reduce its efficacy but also bring about HPD. This is supported by evidence of higher Treg levels in the peripheral blood of non-responders and a recent claim that higher frequency of PD-1^+^ effector T-cells relative to PD-1^+^ Tregs in the tumor predicts positive response to therapy [11,12]. Moreover, increased immunosuppression by Tregs that lack PD-1 signaling has been shown to accelerate tumor development in mice modeled on HPD [13]. Here, we discuss PD-1 blockade enhancing Treg activity and inflicting more detriment on cancer development through HPD. We pay particular attention on the immunoregulatory functions of Tregs that impede anti-tumor immune responses and the pathogenic process that may lead to Treg-driven HPD.

## 2. From Friend to Foe—Regulatory T-Cell (Treg) Induction of Cancer Immune Tolerance

### 2.1. Tregs in Autoimmunity and Cancer

The majority of CD4^+^ Tregs develop in the thymus and constitute about 10% of circulating CD4^+^ T-cells. Tregs play a non-redundant role in immune tolerance and have a master transcription factor, Foxp3, which largely defines the phenotypic and functional characteristics of Tregs. Mutation of FoxP3 gene results in immunodysregulation polyendocrinopathy enteropathy X-linked syndrome in humans and scurfy in mice [14,15,16]. While Tregs are indispensable to protection against autoimmunity, they are undesirable to cancer immunity. A high frequency of Tregs in the tumor usually spells poor prognosis [17]. There are a few exceptions marked by favorable outcomes possibly as a result of Tregs responding but failing to contain strong anti-tumor responses [18]. A key notion is that the more immunogenic tumors are, the more they may be subjected to Treg immunosuppression. This is corroborated by numerous animal studies showing greatly reduced tumors after depleting Tregs or rendering Tregs defective in function [19,20,21]. It is also worth mentioning that selective elimination of intra-tumoral Tregs is sufficient to bolster cytotoxic killing of tumor cells without perturbing the systemic immune system. Using a technique called photodynamic therapy, this was demonstrated by aiming a laser beam at tumors to deplete only tumor-resident Tregs that were pre-bound with a photosensitizer-conjugated antibody against CD25, a dominant Treg surface marker [22]. A similar result from deleting glucocorticoid-induced tumor necrosis factor receptor (GITR)-expressing Tregs would have added more credence, on the grounds that anti-GITR treatment strongly reversed the growth of advanced tumors [23].

### 2.2. Recruitment of Tregs into Tumor

Several chemokine receptors and their partner chemokines have been implicated in the recruitment of Tregs to tumors. CCR4 emerged as a prime candidate after it was found to be expressed in Tregs of human ovarian cancer containing substantial amounts of its ligand, CCL22 [24]. With more precise analyses of the Treg compartment, CCR4 was later found to be present almost exclusively on activated, but not naïve, Tregs [25]. It was then no coincidence that anti-CCR4 treatment in patients with adult T-cell leukemia-lymphoma evoked strong tumor antigen-specific CD8^+^ T-cell response. This was in support of CCR4 blockade reducing tumor-infiltrating Tregs and enhancing anti-tumor immunity especially when combined with tumor vaccines [26,27]. 

In human primary breast cancer, Tregs appear to have a peculiar trafficking pattern as they are recruited by CCL22 to only the border of tumor not within [28,29]. The retention of Tregs at the boundary could be attributed to downregulation or internalization of CCR4 upon binding to CCL22, resulting in cessation of chemotaxis. Tregs in this region are highly activated and proliferative [29]. They may tolerize effector T-cells before their entry into tumor. It would be of interest to assess possible effects of anti-PD-1 treatment on the tumor sentry duties of these Tregs. 

In pancreatic and squamous cell carcinoma, the CCL5:CCR5 axis serves as the main highway for Tregs to infiltrate into tumors [30,31]. Reducing CCL5 production by tumor cells or blocking CCR5 on Tregs significantly lessens the presence of Tregs in tumors. More importantly, CCR5 is preferentially expressed on CD4^+^Foxp3^+^ Tregs over CD4^+^Foxp3^−^ T-cells in healthy individuals and more so in cancer patients [30]. This pattern of expression can be retrieved easily from cells in the peripheral blood and is, therefore, a viable and convenient biomarker.

Besides CCR4 and CCR5, other notable chemokine receptors include CCR8, CXCR3 and CXCR6. These were recently identified as signature genes of tumor-infiltrating Tregs through large-scale mining of Treg transcriptomes from various tumor models and tissues (e.g., colon and spleen) cross-checked between species (humans and mice) and with datasets from the human genome atlas [32]. Interestingly, in vivo CRISPR-Cas9 screen revealed that Tregs deficient in CXCR3, but not CCR8 and CXCR6, were reduced in tumors [32]. This suggests that CCR8 and CXCR6 do not participate in steering Tregs to tumors but may be upregulated by tumor-specific conditions. CCR8, in particular, has been identified as a promising target for specific depletion of intra-tumoral Tregs. Mice administered with anti-CCR8 antibody were shown to have diminished tumors that contained fewer Tregs and more inflammatory cell infiltrates [33]. Two questions that ought to be addressed henceforth are the main mechanism-of-action which CCR8^+^ Tregs employ to create an immunosuppressive environment and the location in tumors where they reside to do so. Additionally, determining the relative expression of PD-1 in CCR8^+^ Tregs and their response to PD-1 blockade would be insightful.

### 2.3. Metabolic Reprogramming of Tumor-Infiltrating Tregs

The tumor microenvironment (TME) is hypoxic and low in glucose. This presents a challenge to tumor-infiltrating T-cells which depend on glycolysis as their primary metabolic machinery. Tregs, however, have an intrinsic advantage with FoxP3 downregulating c-Myc to enable coupling to oxidative phosphorylation (OXPHOS) [34]. As opposed to other T-cells, Tregs display heightened OXPHOS underscored by higher mitochondrial activity and reactive oxygen species [35,36]. A key mediator of OXPHOS in Tregs is liver kinase B1 (LKB1). Tregs deficient in LKB1 are metabolically and functionally impaired owing to dysfunctional mitochondria and dysregulated β-catenin signaling that imposes high PD-1 expression [37]. Blocking PD-1 restored Treg suppression, indicating differential metabolic requirements by Tregs as they transit from one functional state to another.

The versatility of Tregs to adapt to the TME is also underpinned by increased expression of the mitochondrial enzyme, carnitine palmitoyltransferase 1a, which orchestrates the transport of acyl-CoA into mitochondria for fatty acid oxidation (FAO) [38,39]. Hence, Tregs can turn to lipids as a secondary source of energy. Several studies are supportive of fatty acid synthesis and FAO providing a metabolic advantage to Tregs over other T-cells within the tumor [40,41]. In patients with gastric cancer, resistance to PD-1 blockade therapy was traced to a mutation in tumor cells that elevated fatty acid production [42]. This favored Treg survival and prevalence, and was deduced to undermine the effectiveness of anti-PD-1 therapy. 

## 3. Tumor-Associated Immunosuppressive Mechanisms of Tregs

**Figure 1:**
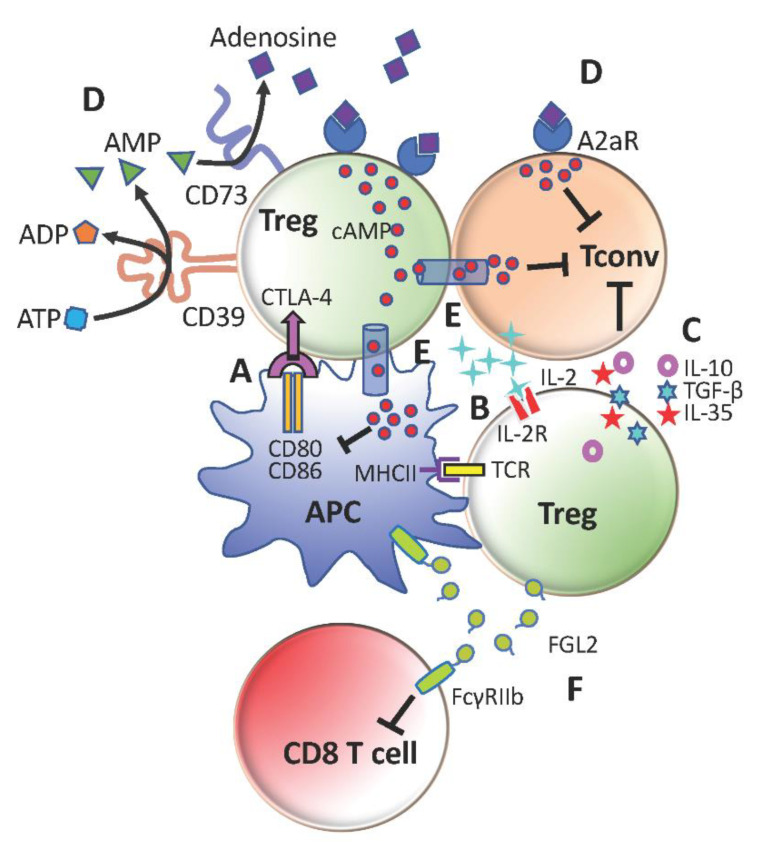
Immunosuppressive mechanisms of regulatory T-cells (Tregs). (**A**) Cytotoxic T-lymphocyte-associated protein 4 (CTLA-4)-mediated downregulation of CD80 and CD86 on antigen-presenting cells (APCs) to limit conventional CD4^+^ T-cells (Tconv) activation. (**B**) Compete with Tconv cells for IL-2 to restrict Tconv survival and proliferation. (**C**) Secretion of anti-inflammatory cytokines TGFb, IL-10 and IL-35 to promote Tconv exhaustion and repress Tconv effector functions (**D**) Generation of adenosine with CD39 and CD73 to suppress Tconv cells and further stimulate Tregs through A2aR (**E**) Transfer of cAMP to inhibit activation of Tconv cells and APCs (**F**) Production of FGL2 to inhibit CD8^+^ T-cells and APCs through FcγRIIb.

Tregs suppress conventional CD4^+^ T-cells (Tconv) by multiple cell-contact and bystander mechanisms (Figure 1). They essentially ‘bubble wrap’ Tconv cells under several layers of regulatory control. This is an efficient and sustainable strategy for a minor to contain a major.

### 3.1. Withhold and Tolerize Antigen-Presenting Cells

Tregs are highly efficient in preventing Tconv cells from accessing antigen-presenting cells (APC). With increased mobility and expression of the integrin, leukocyte function-associated antigen-1 (LFA-1), Tregs form stable and long-lasting aggregates with dendritic cells (DC), thereby restraining DCs from Tconv cells [43]. This is facilitated by an adenosine gradient toward Tregs. Adenosine can serve as a chemoattractant molecule to DCs by stimulating actin cytoskeleton reorganization that initiates motility [44]. As such, Tregs are poised to outcompete Tconv cells for DCs since they express higher levels of CD39 and CD73 necessary for producing adenosine [45,46].

During contact, Tregs confiscate the co-stimulatory molecules, CD80 and CD86, from APCs through cytotoxic T-lymphocyte-associated protein 4 (CTLA4)-mediated trans-endocytosis [47]. This proved to be vital for immune tolerance after it was observed that mice with Treg-specific deficiency of CTLA4 developed overt autoimmunity [48]. Similarly, mice with CTLA4-sufficient Tregs developed larger tumors than those with CTLA4-deficient Tregs. However, it is still unclear whether the increased capacity of Tregs in modulating CD80/86 is solely down to them constitutively expressing high amounts of CTLA4 or dependent on other factors as well. The duration of APCs remaining tolerized and their readiness to become immunocompetent again after Tregs depart are also not known. Answers to these questions and whether they apply to tumor cells as well would provide more clarity on the sustenance of cancer immune tolerance by Tregs. For example, in advanced tumors where tumor cells tend to evade anti-tumor responses by downregulating major histocompatibility complexes (MHC), minimizing CD80/86 on tumor cells may be redundant as APCs remain the only targets of Tregs. Hence, prompt action is paramount to any therapy that intend to control this aspect of Treg cancer immunosuppression or it may be futile in later stages.

### 3.2. Compete with Conventional CD4^+^ T-Cells (Tconv) for Interleukin-2 (IL-2)

Both Tregs and Tconv cells require IL-2 for survival. By comparison, Tregs have a stronger need for IL-2, as shown by the rapid disappearance of Tregs when IL-2 neutralizing antibody was infused into mice [49]. The increased dependence of Tregs on IL-2 may be because of their inherent affinity for self-antigens to protect against autoimmunity, which renders them constantly primed and active in proliferation [50]. This is compounded by the inability of Tregs to produce IL-2 due to Foxp3 itself acting as an IL-2 transcription repressor and its extra-nuclear sequestration of the transcription activators, acute myeloid leukemia 1 (AML1) and nuclear factor of activated T-cells (NFAT) [51,52]. Foxp3, on the other hand, promotes the expression of CD25 (IL2Rα) to form the high affinity IL-2 receptor heterotrimeric complex (IL2Rαβγ) [52,53]. This offers Tregs a competitive edge over Tconv cells for binding to IL-2. In essence, Tregs rely on alternative sources of IL-2 (e.g., Tconv cells) and may starve Tconv cells of IL-2 even from their own production [54]. Hence, IL-2 is deemed to be pivotal to immune homeostasis and an abundance of Tregs in the tumor could exacerbate the constraint of IL-2 limitation on Tconv cells.

### 3.3. Secrete Anti-Inflammatory Cytokines

Tregs produce transforming growth factor β (TGFβ) and IL-10 mainly to modulate Th1 responses. As a Th1 inhibitor, TGFβ activates its receptors, TGFβRI/II, on Tconv cells to counteract the Th1-inducing effects of IFNγ through downregulation of two key Th1 transcription factors namely, T-bet and IFN regulatory factor 1 [55,56]. TGFβ is also required by Tregs themselves to suppress Th17-induced colon inflammation [57]. Unfortunately, the role of Th17 cells in cancer is still controversial, therefore the relevance of this aspect of Treg cancer immunosuppression remains questionable [58].

Although Tregs are major producers of IL-10, the immunomodulatory effects of Treg-derived IL-10 is believed to be rather selective. This has been documented in mice with conditional deletion of the genes, *IL-10* and *Prdm1*, in Tregs. *Prdm1* encodes for the transcription factor, Blimp-1, that is essential for naïve Tregs to differentiate into IL-10-secreting Tregs. In both models, mice manifested colitis as the only severe form of inflammatory disease and rarely developed systemic autoimmunity [59,60]. Its effect on cancer is variable but could become more potent when combined with another cytokine, IL-35, as described below.

The IL-35 molecule is a heterodimer composed of two subunits: Ebi3 and IL12α [61]. IL-35^+^ Tregs are a distinct population from IL-10^+^ Tregs and are independent of Blimp-1 [62]. The former are mostly found in lymphoid organs while the latter in peripheral tissues. Treg-derived IL-35, like IL-10, regulates Tconv cells. This was validated in experimental colitis, which Ebi3^−/−^ and IL12α^−/−^ Tregs failed to rescue [61]. The same was true in vitro for Ebi3^−/−^ and IL12α^−/−^ Tregs exerted less suppression on Tconv proliferation compared to wild type Tregs. Within tumors, both IL-35^+^ and IL-10^+^ Tregs represent sizeable fractions. Mice with compound deficient (IL-35^−/−^IL-10^−/−^) Tregs developed smaller tumors compared to mice with either IL-35^−/−^ or IL-10^−/−^ Tregs [63]. Analyses of gene profiles from Tconv cells that had been exposed to these Treg subtypes proposed that IL-35^+^ Tregs promoted Tconv exhaustion whereas IL-10^+^ Tregs repressed anti-tumor effector functions of Tconv cells [63,64]. On top of their discrete suppressive contributions, both IL-10- and IL-35-secreting Tregs can synergize to mastermind the conversion of activated Tconv cells into immunomodulatory IL-35-producing Tconv cells capable of thwarting anti-tumor immunity [65].

### 3.4. Regulate Extracellular Adenosine Triphosphate (ATP) and Produce Immunosuppressive Adenosine

Dual expression of CD39 and CD73 confers on Tregs two key functions; first in preventing Tconv cells from becoming activated by adenosine triphosphate (ATP) and second in suppressing Tconv cells through adenosine [46]. CD39 and CD73 are ectonucleotidases expressed mainly on the cell surface to hydrolyze ATP/ADP to AMP and AMP to adenosine, respectively [66]. CD39 has 2 transmembrane domains that are integral to its enzymatic activity [67,68]. As for CD73, it is anchored to the membrane by a single glycosylphosphatidylinositol residue [69]. Homodimerization coupled with binding to 2 zinc ions must occur for CD73 to become catalytically active [70,71]. When cleaved by phospholipase C, CD73 can be released as soluble form into the extracellular space.

Under steady-state, ATP is mostly confined within cells (3–10 mM) and is virtually absent in the external environment (~10 nM) [72]. A net increase in extracellular ATP can ensue from necrotic cells or inflamed cells that release intracellular ATP through vesicular exocytosis and membrane transporters and channels [73]. This is especially prevalent in ischemic and hypoxic settings such as in the tumor [74]. There are two classes of ATP-binding P2 purinergic receptors, P2XR and P2YR. Both are present on DCs and monocytes whereas only P2XR is expressed on lymphocytes [66]. ATP sensing by P2X1R, P2X4R, P2X5R and P2X7R are known to activate T-cells, with the exception of Tregs which apoptose upon strong ATP interaction with P2X7R [75,76]. For this reason, hydrolysis of ATP by Tregs is quintessential to the homeostasis of Tconv cells.

The immunosuppressive effect of CD73-derived adenosine is mediated through 2 G-protein coupled receptors, Adora2A (A2aR) and Adora2b (A2bR). Between them, A2aR has a higher affinity for adenosine and is constitutively expressed in T-cells [77,78]. A2bR is upregulated in activated Tregs and have a profound influence on their function [79]. When bound to adenosine, both receptors stimulate adenylyl cyclases to generate cyclic AMP (cAMP). cAMP would then trigger protein kinase A (PKA) to block proliferation and cytokine production [80]. Two downstream molecules that are responsible for the inhibitory response are inducible early cAMP repressor (ICER) and exchange protein activated by cAMP (EPAC). Binding of ICER to activator protein-1 (AP-1) and NFAT blocks IL-2 production and cell-cycling [81,82]. The EPAC proteins, EPAC1 and EPAC2, regulate cellular metabolism by converting guanosine triphosphate (GTP) to GDP [83].

During tumor development, Tregs are active in limiting ATP levels and producing adenosine to suppress anti-tumor effector T-cells, as attested by attenuated tumor growth in mice with either CD39-deficient or CD73-deficient Tregs [84,85]. Remarkably, even apoptotic Tregs exploit their own ATP leakage to produce adenosine to suppress anti-tumor immunity, including abrogating the effects of PD-1 blockade [86].

### 3.5. Transfer cAMP into Dendritic Cells (DCs) and Tconv Cells

In contrast to Tconv cells, high cAMP content is integral to Treg function [87]. The more activated Tregs are, the more cAMP accumulates in them [88]. This specialized feature of Tregs coincides with FoxP3-mediated downregulation of the cAMP hydrolyzing enzyme, phosphodiesterase 3b (PDE3b), and upregulation of adenylyl cyclase 9 (AC9) [89,90]. Furthermore, adenosine generated by Tregs feeds an autocrine loop to reinforce cAMP production through the adenosine receptors [91].

When Tregs contact Tconv cells, intercellular gap junctions are formed for cAMP to pass from Tregs into Tconv cells. This direct mode of suppression inhibits IL-2 expression and anergizes Tconv cells [88]. An increase in cAMP may also dampen T-cell receptor stimulation via PKA activation of C-terminal Src kinase (Csk) and even potentiate differentiation of Tconv cells into Tregs in the presence of TGFβ, thus highlighting infectious tolerance as a conceivable case in point [79,80,92].

The exact sequence of events has not been completely elucidated. However, it is plausible that APCs provide the platform for physical interaction between Tregs and Tconv cells that recognize cognate antigens. This was clearly shown in vivo with ovalbumin (Ova)-specific Tconv cells receiving the cell-labeling agent, calcein, from calcein-labeled Ova-specific Tregs only in lymph nodes that drained the Ova-vaccination site but not in non-draining lymph nodes [88]. This may represent a scenario reminiscent of tumor-specific DCs, Tregs and Tconv cells shuttling between the tumor and regional lymph nodes. DCs were, accordingly, found to receive substantially more cAMP from Tregs compared to Tconv cells [93]. Aside from EPAC1 antagonizing PKA and resisting DC maturation, the signaling pathways downstream to cAMP influx into DCs from Tregs is still not well understood [80,94].

In cancer, much of the delivery of cAMP from Tregs to Tconv cells and DCs is expected to take place in regional lymph nodes where they are organized in specific zones for maximal contact. This could prove to be one of the more efficient means of Treg suppression, considering Tconv cells and DCs tolerized in this manner are unlikely to egress and travel to the site of tumor. A good illustration of such Treg-contact suppression can be found in DCs that decline into a state of quiescence shortly after forming strong conjugates with Tregs [95]. This calls for more studies to better understand the importance of cAMP-mediated pathways in tumor immunity.

### 3.6. Produce Fibrinogen-Like Protein 2 (FGL2) to Inhibit FcγRIIB-Expressing Cells

One hitherto underappreciated function of Tregs is the secretion of fibrinogen-like protein 2 (FGL2). As its name implies, FGL2 is a key component of the coagulation cascade and possesses pro-thrombinase activity [96]. It is also known to have immunosuppressive effects as evidenced by autoimmune glomerulonephritis in mice deficient in FGL2 [97].

FGL2 exists in membrane as well as soluble forms. CD4^+^ and CD8^+^ T-cells, most prominently Tregs, produce only soluble FGL2 [96]. Foxp3 in Tregs drives FGL2 expression. Hence, FGL2 has been ascribed as a Treg-signature molecule [89]. Tregs from FGL2-deficient mice are less able to suppress Tconv cells in vitro [97]. The main immunomodulatory action of FGL2 takes place through FcγRIIB in APCs. Like FGL2-deficient mice, FcγRIIB-deficient mice also develop autoimmune glomerulonephritis [98,99]. FcγRIIB is a single-chain receptor that transduces inhibitory signals from its immunoreceptor tyrosine-based inhibition motif (ITIM) to inhibit other activation Fcγ receptors [100]. In DCs, however, binding of FGL2 to FcγRIIB does not activate ITIM [101]. Instead, NFκβ is prevented from upregulating MHCII and CD80, keeping DC maturation in check [102].

Lately, FcγRIIB has also been detected on activated CD8^+^ T-cells, notably more than CD4^+^ T-cells [103]. This is least expected for a lymphocyte population that hardly expresses Fcγ receptors. FGL2-stimulated FcγRIIB^+^ CD8^+^ T-cells have increased caspase 3/7 induction, which compels them to apoptosis [103]. Physiological relevance is underscored by mice with FcγRIIB-deficient CD8^+^ T-cells exhibiting smaller tumors. Going forward, an intriguing proposition that warrants further enquiry is the direct negative impact of Treg-derived FGL2 on anti-tumor CD8^+^ T-cells independent of DCs and Tconv cells. The paucity of anti-tumor immune responses may well be FGL2 produced by Tregs contributing in part to a reduction of tumor-infiltrating CD8^+^ T-cells.

## 4. Tregs ‘Tipped’ by Anti-Programmed Cell Death Protein 1 (PD-1) Turn the Tables on Anti-Tumor T-Cells

### 4.1. PD-1 Inhibitory Signaling

When T-cells become activated, NFATc1 binds to the promoter of PD-1 to initiate transcription [104]. This is augmented on re-stimulation and is counterbalanced by the association of special AT-rich sequence binding protein 1 (Satb1) and nucleosome remodeling deacetylase (NURD) in the enhancer regions of PD-1 [105]. T-cells deficient in Satb1 promptly gain PD-1 and assume dormancy. In tumors, de-repression of PD-1 may occur with TGFβ-induced Smad proteins displacing the Satb1:NURD complex [105]. This could explain the especially high expression of PD-1 in tumor-infiltrating T-cells.

The cytoplasmic domain of PD-1 comprises two tyrosine-based residues, ITIM and immunoreceptor tyrosine-based switch motif (ITSM). Phosphorylation of ITSM recruits SH2-domain containing tyrosine phosphatase 2 (SHP-2) to dephosphorylate adaptor molecules of the TCR complex, such as CD3, zeta-chain-associated protein of 70kDa (ZAP70) and phosphatidylinositol-3-kinase (PI3K) [106]. This process may also relieve Csk from inhibition by SHP-2, allowing Csk to preclude lymphocyte-specific protein tyrosine kinase (Lck) from activating CD3 and ZAP70 [107].

### 4.2. Shortcomings of Anti-PD-1 Cancer Immunotherapy

Among the cancer types treated by PD-1 blockade therapy, Hodgkin’s disease has the highest objective response rate at 87%, while head and neck, gastroesophageal and bladder cancer share the lowest at 15% [108]. The tumor mutational burden ranks high on the list of factors that influence treatment efficacy [109,110]. It can be categorized by mutations that prevail from early tumor cells, termed clonal, or mutations that arise during tumor development, termed ‘subclonal’ [110]. In general, anti-PD-1 therapy is more effective against tumors that contain higher clonal than subclonal mutations. These tumors are likely to have a substantial pool of PD-1^+^ effector T-cells ready to be unleashed against clonal antigens that are ubiquitously expressed on tumor cells. On the other hand, a far from absolute response is expected of tumors with subclonal antigens. Tumor cells that escape from anti-PD-1 immunotherapy may thrive better after the destruction of their more vulnerable neighbors, particularly in a nutrient scarce tumor. This could precipitate tumor progression. Under such a circumstance, unless sufficient effector T-cells are generated against subclonal antigens, anti-PD-1 therapy would be less effective. Tregs could present a major hurdle too.

### 4.3. PD-1 Signaling Inhibits Treg Activity and Blockade of PD-1 Enhances Treg-Mediated Immunosuppression

In patients with glioblastoma multiforme, Tregs with high PD-1 expression are less suppressive and have a transcriptomic profile that bears an exhausted signature [111]. It was, likewise, the case for Tregs in patients infected with hepatitis C [112]. Blocking PD-1 in Tregs isolated from livers of these patients gave rise to increased Treg proliferation and suppression.

Consistent with PD-1 restricting Treg activity, CXCR5^+^ follicular Tregs from PD-1 deficient (PD-1KO) mice were found to be more immunosuppressive in vitro [113]. Correspondingly, adoptive transfer of PD-1KO Tregs into mice susceptible to autoimmune pancreatitis led to better suppression of pathogenic Tconv and CD8^+^ T-cells and better protection of the pancreas compared to the transfer of wild-type Tregs [114]. Reduced type 1 diabetes in non-obese diabetic (NOD) mice with PD-1 deficiency limited to only Tregs provided the strongest affirmation for a repressive role of PD-1 in Treg immunosuppression [115]. Tregs devoid of PD-1 were also shown to have reduced PI3K-AKT signaling that is typical of activated Tregs.

As with the intent of releasing the ‘brake’ on anti-tumor T-cells, PD-1 blockade therapy may still have to circumvent the pro-tumoral Treg barrier. Blockade of PD-1 could have a pronounced impact on Tregs as they constantly engage and receive TCR stimulation from APCs. In a Phase I trial of nivolumab administered to stage III/IV melanoma, an increase in Treg frequency was observed in leukapheresis specimens of non-responders [11]. In addition, animal tumor models resistant to anti-PD-1 became less so with concurrent depletion of Tregs using the anti-CD25 antibody [116].

**Figure 2:**
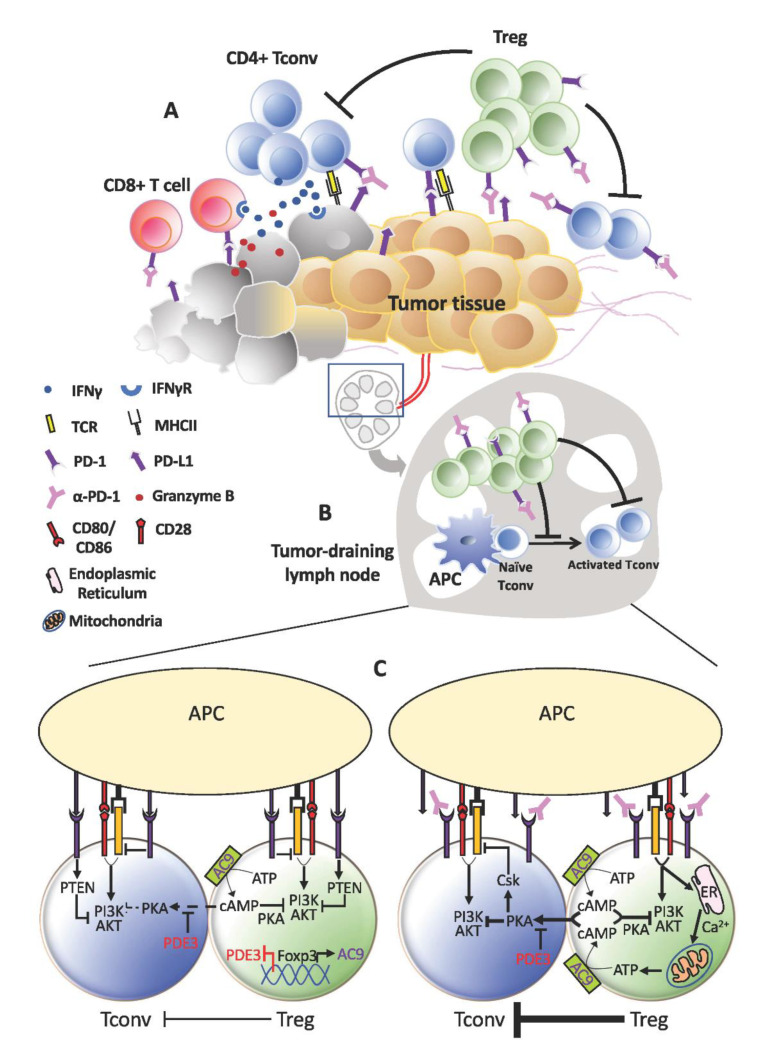
Treg expansion behind hyper-progressive disease (HPD). (**A**) Within the tumor, the anti-tumor effects of programmed cell death protein 1 (PD-1) blockade on effector T-cells may be short-lived due to the activation and expansion of Tregs. This could be even more pronounced in tumors that contain higher proportions of PD-1^+^ Tregs relative to T-effector cells. Consequently, tumor cells that escape continue to thrive and proliferate. (**B**) A similar occurrence may take place in the lymphoid organs where a more active Treg population resists fresh generation of anti-tumor T-effector cells. As anti-tumor immunity wanes, tumor progresses unhindered and may even accelerate in the form of HPD. (**C**) At the cellular level, increased TCR signaling in Tregs devoid of PD-1 signaling may drive increased calcium influx and adenosine triphosphate (ATP) production, which in turn elevates cAMP generation and transfer into Tconv cells. Consequently, TCR stimulation in Tconv cells could still be subdued by the PKA:Csk despite absence of PD-1 inhibitory signals.

More than offsetting tumor-killing effects, the expansion of Tregs that accompanies PD-1 blockade could instigate tumor progression akin to HPD (Figure 2). As previously reported by us, increased Treg proliferation was apparent in advanced gastric cancer patients diagnosed with HPD. We further verified in murine experiments that PD-1 deficiency and blockade in Tregs boosted their immunosuppressive action against even PD-1KO effector T-cells to advance tumor development [13]. Nonetheless, Tregs are unlikely to be the sole cause of HPD. Certain underlying preconditions may advance tumors to the verge of explosive growth before PD-1 blockade escalates the process through Treg activation. These could be present during the ‘equilibrium’ phase, defined as the stage where tumor eradication by effector T-cells is matched by tumor evasion [117]. At this point of inflection, any decrease in effector T-cell activity could benefit tumor cell survival and division. For instance, the metabolic rate of tumor cells may be spurred as more glucose becomes available. This is a reasonable speculation in view of the strict reliance on aerobic glycolysis by tumor cells and effector T-cells. All in all, anti-PD-1 may have less activated PD-1^+^ effector T-cells to target from increased Treg suppression.

## 5. Tracing the Path to Hyper-Progressive Disease (HPD) after Treg Expansion by PD-1 Blockade

While blocking the inhibitory signals of PD-1 increases Treg activation, Tregs may still require IL-2 for robust proliferation. A study by Asano and co. showed that Tregs, but not non-Treg T-cells, expanded after IL-2 was administered at a low dose to mice [118]. The number of Tregs rose sharply after treatment with anti-PD-1. This was followed by a quick return to baseline level due to Treg apoptosis. A similar trend was observed in PD-1KO mice. 

In the context of anti-PD-1 cancer immunotherapy, Treg expansion may be sustained by transient release of IL-2 from effector T-cells in the initial phase. In time, IL-2 secretion could subside as effector T-cells become increasingly suppressed by Tregs. Discontinuation of IL-2 production in effector T-cells may also be brought forth by increased expression of other co-inhibitory molecules, such as Tim-3 (T-cell immunoglobulin and mucin domain-3) and Lag-3 (lymphocyte-activation gene 3) [119,120]. This could expedite exhaustion in effector T-cells and prevent them from re-committing to the killing of tumor cells.

Although the apoptotic rate of Tregs during anti-PD-1 treatment is not known, increased Treg apoptosis could potentiate tumor progression. This is based on the strong immunosuppressive effect of dying Tregs through adenosine production as mentioned above [86]. However, it is unlikely for extra adenosine to cause HPD simply by acting against effector T-cells that are already largely suppressed. Rather, adenosine has several pro-tumoral properties that could steepen the growth trajectory of tumor cells. Among them are improving tumor cell survivability through the regulation of anti- (Bcl2) and pro- (p53 and Bax) apoptotic genes and fostering proliferation through the induction of relevant signaling pathways (e.g., Akt and Erk1/2) as well as cell-cycle factors (e.g. cyclins A, B, D and E) [121]. Beyond these, adenosine may raise the chance of tumor malignancy by weakening the adhesion and stability of primary tumors and enhancing angiogenesis that provides conduits for tumor cells to emigrate and invade other tissues and organs [121,122,123]. Nevertheless, there are occasions where adenosine is linked to tumor growth arrest [121]. Variations in response to adenosine may stem from the subtypes (A1R, A2aR, A2bR, A3R) and levels of adenosine receptors expressed in tumor cells.

It is imperative not to overlook the possibility of Treg expansion causing HPD. It is equally imperative not to assume any degree of Treg expansion leads to HPD. Hypothetically, minor Treg expansion may only give rise to non-response to therapy or moderate tumor progression. Major Treg expansion, which may be contingent to IL-2 availability, and a subsequent ‘tsunami’ of Treg apoptosis plus adenosine production is more likely to culminate in HPD. Further work is needed to uncover the pathogenesis of Treg-mediated HPD. This is a difficult undertaking given the extremely short time frame of HPD.

At present, tumors assessed in accordance with RECIST1.1 with growth rate of more than two-fold at first examination compared to pre-therapy would qualify as HPD. Stricter criteria have been proposed to classify and distinguish HPD from other forms of cancer progression [124]. Some recommendations are the inclusion of time to treatment failure (TTF), measurement of the longest tumor dimension relative to time to better represent growth kinetics, additional radiological scans and detection of tumor metastasis [8,10,125,126]. Perhaps, these stringent guidelines may expose differences between HPD cases that are dependent and independent of Treg expansion. One may expect HPD driven by Treg expansion to have shorter TTF and prone to metastasis. Additionally, the growing list of HPD biomarkers can be stratified by the presence of Tregs in tumors [127]. This may assist in identifying correlations between Tregs and certain tumor-associated features that have a higher propensity for HPD development. Dissecting Treg populations into viable and apoptotic Tregs and analyzing the variety of adenosine receptors in tumor biopsies would be desirable in this respect.

## 6. Conclusions and Perspectives

PD-1 blockade is certainly a feasible method to revive dormant and exhausted anti-tumor Tconv and CD8^+^ T-cells. On the flip side, Tregs can also become more active and exert greater suppression on them as well as those undergoing activation from the naïve state. Depending on the degree of tumor immunity inhibited, tumor cells would be able to proliferate unchallenged, increasing the likelihood of HPD.

**Figure 3:**
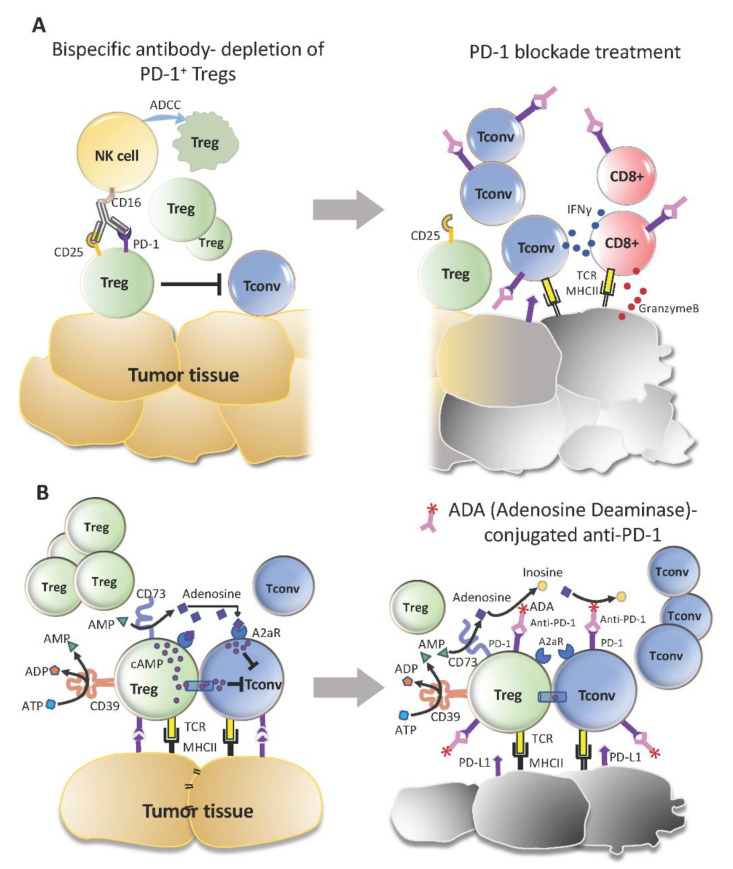
Possible treatment strategies for PD-1 blockade cancer immunotherapy to circumvent Tregs. (**A**) PD-1^+^ Tregs may first be depleted by antibodies that are bispecific for CD25 and PD-1. Subsequent treatment with PD-1 blocking antibody would likely enhance only Tconv cells and CD8^+^ T-cells. (**B**) The generation of adenosine by Tregs may not only suppress Tconv cells but also augment Treg activity. Hence, an obstacle to anti-PD-1 therapy. This could be addressed using anti-PD-1 antibodies conjugated with the enzyme, adenosine deaminase, which deaminates adenosine to inosine. A secondary effect could also be achieved from reduced cAMP generation in Tregs and transfer into Tconv cells.

The acuteness of HPD prevents any meaningful intervention to ameliorate it in time to avoid fatality. Therefore, due consideration has to be taken at the individual level to ascertain the suitability of anti-PD-1 therapy. To this end, a series of tests can be performed beforehand to assess the immune profile in each patient and predict its response to PD-1 blockade. This underlines the importance of searching for reliable clinical and biological markers that would unveil tell-tale signs of HPD. According to a recent report, females may be more at risk of developing HPD [128]. If true, further analyses would be required to determine the involvement of Tregs, as implied by their role in regulating inflammation within visceral adipose tissue in response to sex-hormones [129]. The challenge with Tregs is the rarity of Treg-specific markers, let alone any unique to the PD-1-expressing Treg subset. Alternatively, bispecific antibodies that recognize CD25 and PD-1 can be designed for the depletion of PD-1^+^ Tregs (Figure 3). This would minimize their presence and condition the tumor for PD-1 blockade on PD-1^+^ effector T-cells rather than Tregs. Meanwhile, close monitoring is key to an early warning system that would sound the alarm for weaning patients off anti-PD-1 before it causes irreversible damage.

To conclude, the resilience of Tregs in cancer is proving to be a thorn to anti-PD-1 immunotherapy. From the current outlook, anti-PD-1 may have to evolve in combination with novel Treg depletion strategies or therapies that reduce Treg function for better results and minimal shortfalls.
